# Increased ethanol accumulation from glucose via reduction of ATP level in a recombinant strain of *Saccharomyces cerevisiae* overexpressing alkaline phosphatase

**DOI:** 10.1186/1472-6750-14-42

**Published:** 2014-05-15

**Authors:** Marta V Semkiv, Kostyantyn V Dmytruk, Charles A Abbas, Andriy A Sibirny

**Affiliations:** 1 Institute of Cell Biology, NAS of Ukraine, Drahomanov Street, 14/16, Lviv 79005, Ukraine; 2 University of Rzeszow, Zelwerowicza 4, Rzeszow 35-601, Poland; 3 Archer Daniels Midland Co Research Center, 1001 N Brush College Rd, Decatur, IL 62521, USA

**Keywords:** Baker’s yeasts, Ethanol production, *PHO8*, Alkaline phosphatase, ATP content, Biomass accumulation

## Abstract

**Background:**

The production of ethyl alcohol by fermentation represents the largest scale application of *Saccharomyces cerevisiae* in industrial biotechnology. Increased worldwide demand for fuel bioethanol is anticipated over the next decade and will exceed 200 billion liters from further expansions. Our working hypothesis was that the drop in ATP level in *S. cerevisiae* cells during alcoholic fermentation should lead to an increase in ethanol production (yield and productivity) with a greater amount of the utilized glucose converted to ethanol. Our approach to achieve this goal is to decrease the intracellular ATP level via increasing the unspecific alkaline phosphatase activity.

**Results:**

Intact and truncated versions of the *S. cerevisiae PHO8* gene coding for vacuolar or cytosolic forms of alkaline phosphatase were fused with the alcohol dehydrogenase gene (*ADH1*) promoter. The constructed expression cassettes used for transformation vectors also contained the dominant selective marker *kanMX4* and *S. cerevisiae* δ-sequence to facilitate multicopy integration to the genome. Laboratory and industrial ethanol producing strains BY4742 and AS400 overexpressing vacuolar form of alkaline phosphatase were characterized by a slightly lowered intracellular ATP level and biomass accumulation and by an increase in ethanol productivity (13% and 7%) when compared to the parental strains. The strains expressing truncated cytosolic form of alkaline phosphatase showed a prolonged lag-phase, reduced biomass accumulation and a strong defect in ethanol production.

**Conclusion:**

Overexpression of vacuolar alkaline phosphatase leads to an increased ethanol yield in *S. cerevisiae*.

## Background

Alcoholic fermentation represents the largest application of the yeast *Saccharomyces cerevisiae* in the field of industrial biotechnology. In 2011, worldwide fuel ethanol production reached 84.6 billion liters [1]. With increased ethanol use as a biofuel and continued growth in its production for distilled beverages, the worldwide annual production of ethanol will exceed in 2014 100 billion liters. Due to economic and environmental reasons, exponential growth in the production of fuel ethanol was observed over the past decade [2]. Though lignocellulose is considered to be one of the most promising feedstocks for production of fuel ethanol, its current industrial production relies heavily on fermentation of traditional feedstocks such as sucrose (derived primarily from sugarcane or sugar beets) and glucose obtained from starchy materials (corn, wheat, barley, potatoes etc.). Therefore the construction of the strains of *S. cerevisiae* with elevated ethanol production (yield and productivity) from glucose is of great academic and industrial interest. We decided to reach this goal by manipulation of the ATP content in the cell.

The yeast *S. cerevisiae* catabolizes glucose via the Embden-Meyerhof-Parnas (EMP) pathway which yield anaerobically 2 moles ATP per mole of consumed glucose. The efficiency of this pathway for anabolic processes is low with a maximal biomass yield of around 7% and an ethanol yield in the range of 90% - 93% from the glucose consumed [3]. Even a slight shift of this ratio in favour of greater ethanol yield from dextrose, can provide an additional several millions litres of ethanol to the worldwide production annually. In contrast to *S. cerevisiae*, the bacterium, *Zymomonas mobilis,* ferments glucose through Entner-Doudoroff (ED) pathway. This pathway provides only 1 mole of ATP per mole of glucose, and consequently directs only 3% of glucose to cell biomass achieving ethanol yield of up to 97% of the possible theoretical value [4]. This indicates that lowering the ATP yield during alcoholic fermentation increases ethanol yield with reduced substrate conversion to cell mass. Fast fermentation of glucose to ethanol is another important advantage of *Z. mobilis* over *S. cerevisiae*[4,5]. Attempts to substitute *S. cerevisiae* by *Z. mobilis* for the production of industrial ethanol were considered to be a promising approach to increase ethanol yield. However, *Z. mobilis* has several serious drawbacks which hamper its industrial use and these consist of: (i) a very narrow substrate range (only glucose is efficiently fermented whereas sucrose fermentation is hardly proceeds with low yield), (ii) natural auxotrophy for lysine, methionine and some vitamins, (iii) non-GRAS status, which prevents using cellular biomass as a feed additive, (iv) requirement for a higher pH for growth [6,7]. Furthermore, the technology of yeast cell utilization for alcoholic fermentation is well developed whereas the use of bacterial cells for ethanol production is far less common. Thus, it looks like construction of the yeast strains which produce less ATP during alcoholic fermentation would be a better approach to increase ethanol yield. These new yeast strains would combine all of the possible advantages of yeast with the high ethanol yield of *Z. mobilis*. There could be several approaches to achieve this goal, for example: (i) substitution of EMP pathway in yeast by ED pathway from *Z. mobilis* or other bacteria possessing genes of the pathway; (ii) increasing the activity of the enzymes involved in generation of futile cycles; (iii) the introduction of heterologous genes encoding for energy-consuming plasma membrane glucose symporters or (iv) construction of recombinant strains with elevated activity of ATPase of other ATP-degrading enzymes [8].

The first approach to express *Escherichia coli* ED dehydratase and ED aldolase genes *edd* and *eda* in a phosphofructokinase deficient mutant of *S. cerevisiae* has been published [9]. The engineered strains grew and fermented glucose to ethanol, though activities of ED dehydratase and ED aldolase were not reported. It is known that quite often prokaryotic enzymes display low or no activity in *S. cerevisiae* hosts [10], which is most likely caused by improper folding or instability of the expressed bacterial protein in yeast. In addition, there are difficulties in NADP regeneration in the yeast engineered to possess ED pathway since NADPH produced in the glucose-6-phosphate dehydrogenase reaction, cannot be re-oxidized via alcohol dehydrogenase reaction and yeast does not have NADH/NADPH transhydrogenase activity [11,12].

Alternative approaches to lower ATP level in yeast cell and increase ethanol yield could rely on the activation of some cytosolic ATPases, other ATP hydrolyzing enzymes or via the induction of the some kinds of futile cycles to dissipate cellular pool of ATP, e.g. bacterial or modified yeast fructose-1,6-bisphosphatase [13-15]. There are also published data on decrease of cellular pool of ATP and activation of alcoholic fermentation through overexpression of the soluble part (F_1_) of H^+^-ATPase or a portion of F_1_ exhibiting ATPase from different origin in *S. cerevisiae*[16]. Similar results were obtained after overexpression of *PHO5* coding for acid phosphatase which is non-specific enzyme hydrolyzing also ATP [15].

In earlier work, we carried out a successful attempt to decrease intracellular ATP level by overexpression of 5’ part of the *S. cerevisiae SSB1* gene encoding cytosolic ATPase domain and by the heterologous gene *apy* encoding apyrase from *E. coli*[17]. In this paper, we describe a new method to lower cellular ATP and increase ethanol yield and productivity during glucose fermentation by overexpression of *S. cerevisiae* intact *PHO8* gene which codes for alkaline phosphatase.

## Results and discussion

### Overexpression of the vacuolar forms of alkaline phosphatase in BY4742 laboratory strain

*S. cerevisiae* unspecific alkaline phosphatase (phosphomonoesterase), located in the vacuole, encoded by the gene *PHO8,* catalyses the dephosphorylation of many different compounds including ATP [18,19]. Therefore this enzyme may operate as non-specific ATPase. We hypothesized that an increase in alkaline phosphatase activity will reduce intracellular ATP level and thus enhance ethanol production under anaerobic conditions. Several factors should be taken into account to increase specific activity of the target enzyme in yeast. Among these are the copy numbers used for the gene of interest, choice of promoter which controls expression level, stability of the synthetized mRNA, and enzyme feedback inhibition. We have demonstrated that the *ADH1* promoter for the gene encoding alcohol dehydrogenase that we used in this work, is very strong and is two-fold activated under the conditions of alcoholic fermentation [O. Kurylenko, K. Dmytruk, A. Sibirny, unpublished observations]. Therefore the *ADH1* gene promoter was used for *PHO8* gene overexpression. Additional activation of *PHO8* overexpression can be achieved by multicopy genomic integration. In order to insert a high copy number of the *PHO8* gene into the yeast genome, an integrative plasmid containing δ sequences was constructed. The *S. cerevisiae* δ sequences are the long terminal repeats of the retro-transposons Ty1 and Ty2 with an estimated 425 δ sequences dispersed throughout the yeast genome. It was shown that the use of vectors containing δ sequences can provide tandem multicopy integration in one or several sites of yeast genomic DNA via homologous recombination [20]. The δ sequences-based plasmid pUC57-delta1_2-ADHpr-PHO8-CYCt-kanMX harboring *PHO8* gene under the control of *ADH1* promoter was constructed as described in Materials and Methods and subsequently transformed into *S. cerevisiae* laboratory strain BY4742 (Table 1).

**Table 1:** Strains and plasmids used in the present investigation

**Strains**	**Genotype**	**Reference**
BY4742	*MAT*α, *his3*Δ*1*, *leu2*Δ*0*, *lys2*Δ*0*, *ura3*Δ*0*	[21]
BY4742/Pho8vac	BY4742, P_ADH_-PHO8-kan^r^	This study
BY4742/Pho8cyt	BY4742, P_ADH_-PHO8_trunc-kan^r^	This study
AS400	Wild type strain commercially used in bioethanol production at Archer Daniels Midland Company (Decatur, IL, USA)	This study
AS400/Pho8vac	AS400, P_ADH_-PHO8-kan^r^	This study

**Figure 1:**
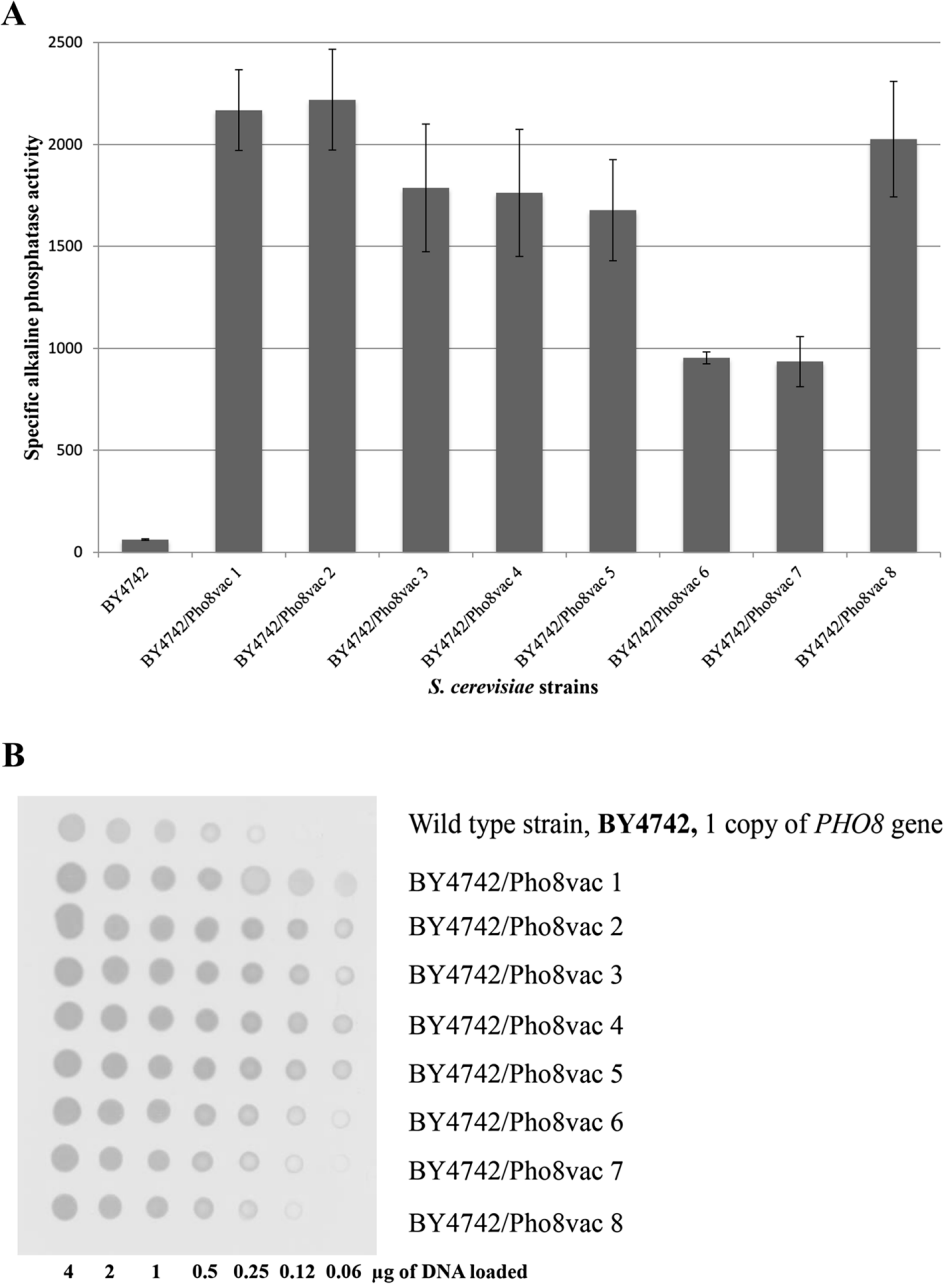
Specific alkaline phosphatase activity in nmoles of product/mg of prot.*min (A) and estimation of integrated expression cassette copy number by dot-blot hybridization (B) in the *S. cerevisiae *transformants and control strain. BY4742 – recipient strain; BY4742/Pho8vac – recombinant strains bearing plasmid pUC57-delta1_2-ADHpr-PHO8-CYCt-kanMX. Alkaline phosphatase activity is shown in nmoles of product/mg of prot.*min. For dot-blot hybridization genomic DNA was isolated from WT and recombinant strains, diluted and loaded onto nitrocellulose film. Amounts of loaded DNA is shown under the photo. *PHO8* gene was used as a probe.

The specific alkaline phosphatase activity of the selected recombinant strains, derivative of BY4742, was assayed. Among 8 tested transformants, six strains were characterized by 30–40 times higher specific phosphatase activity relative to the wild-type strain BY4742 (Figure 1A, strains 1–5 and 8). The data confirm that the constructed expression vector provided multi-copy integration into the genome of *S. cerevisiae*.

Genomic DNA isolated from the recombinant strains was subjected to dot-blot hybridization to estimate plasmid copy number harbouring target gene, integrated into the genome. Gene *PHO8* was used as a probe (Figure 1B). The recombinant strains contained 1–9 additional copies of *PHO8* gene with a good correlation between *PHO8* gene copy number and specific alkaline phosphatase activity was observed.

**Figure 2:**
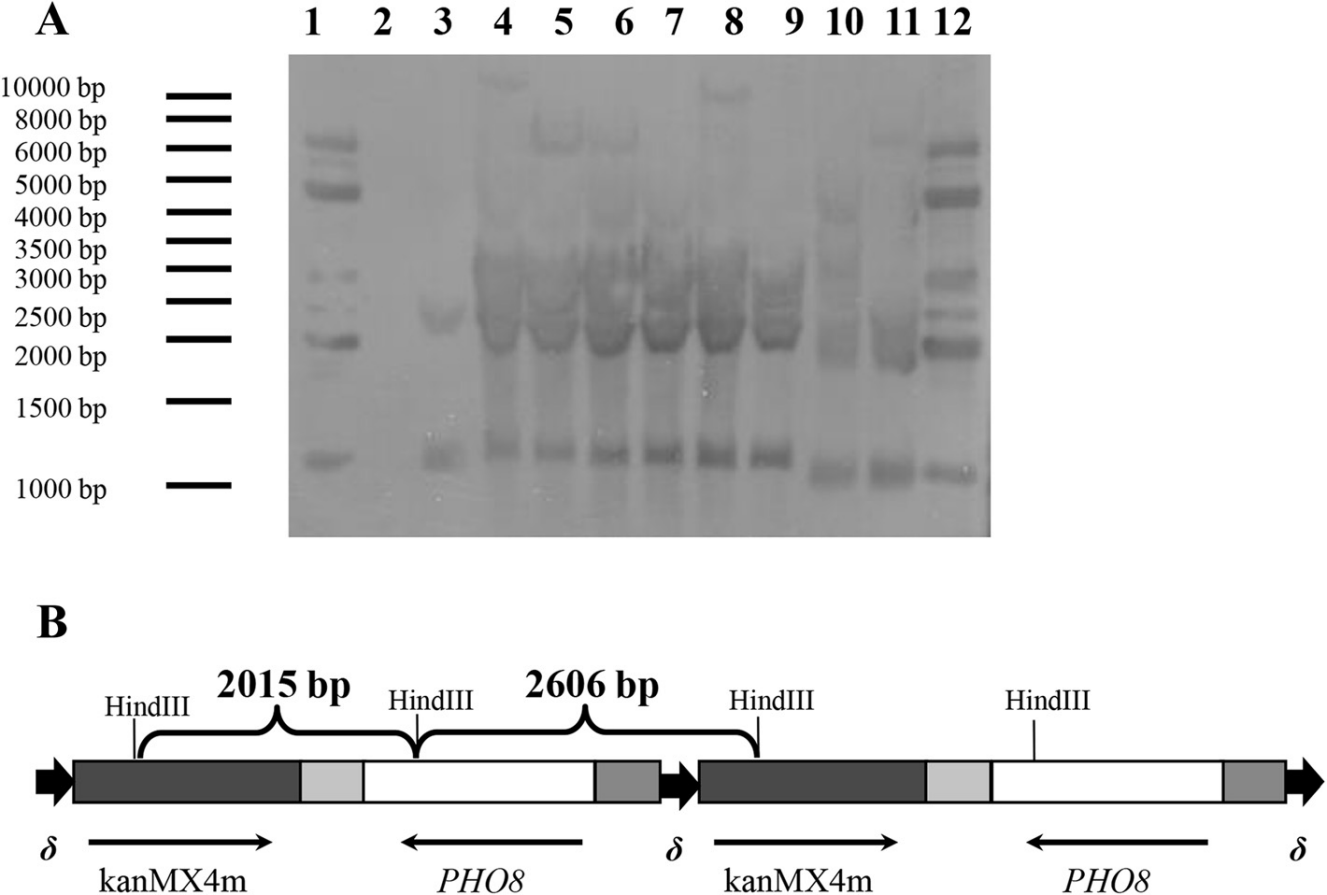
**Analysis of expression cassette integration pattern. (A)** Southern hybridization. *PHO8* gene was used as a probe. HindIII was used for genomic DNA restriction. 1, 12 – plasmid pUC57-delta1_2-ADHpr-PHO8-CYCt-kanMX; 2 – ladder; 3 - wild type strain, BY4742; 4–11 – BY4742/Pho8vac – recombinant strains containing vector pUC57-delta1_2-ADHpr-PHO8-CYCt-kanMX; **B**) “head-to-tail” conformation of vector integration.

Southern-hybridization was performed to analyse the vector integration pattern of the tested strains (Figure 2A). Total genomic DNA from wild type and recombinant strains were HindIII digested and hybridized with a labelled *PHO8* gene. The constructed δ sequences-based vector had tandem multi-copy integration in up to three sites of yeast genomic DNA in the so called “head-to-tail” conformation (Figure 2B), which is in good agreement with previously published results [20].

A selected recombinant strain with substantially enhanced level of specific alkaline phosphatase activity showed small defects in biomass accumulation and intracellular ATP level relative to that of the wild-type strain (Table 2). Ethanol productivity of the wild-type strain BY4742 was 0.79 g/L/h or 0.30 g/g of biomass/h, whereas the recombinant strain BY4742/Pho8vac, had a much higher productivity: 0.92 g/L/h or 0.34 g/g of biomass/h during glucose alcoholic fermentation. The *PHO8*-overexpressing recombinant strain accumulated 13% more ethanol as compared to the parental strain BY4742 (Table 2). The data provide proof to our initial hypothesis that the overexpression of alkaline phosphatase can be used to increase ethanol synthesis during glucose alcoholic fermentation.

**Table 2:** **Growth rate, ATP level, AP activity, ethanol productivity and yield of *S. cerevisiae *** **transformants and control strains**

**Strain**	Specific growth rate, g/L/h^*^	ATP, μmoles of ATP/mg dry cell weight^**^	Alcaline phosphatase activity, nmoles of product/mg of prot.*min.^*^	Ethanol productivity^*^		Ethanol yield g/g of consumed glucose^*^
				**g/L/h**	**g/L/g of biomass/h**	
BY4742	0.031 ± 0.002	7.95 ± 0.10	85.2 ± 3.3	0.79 ± 0.014	0.30 ± 0.006	0.379 ± 0.007
BY4742/Pho8vac	0.032 ± 0.001	7.83 ± 0.06	1948.3 ± 175.3	0.92 ± 0.019	0.34 ± 0.007	0.442 ± 0.009
BY4742/Pho8cyt	0.025 ± 0.001	7.56 ± 0.08	1832.4 ± 137.4	0.42 ± 0.004	0.18 ± 0.002	0.202 ± 0.002
AS400		8.93 ± 0.15	45.3 ± 2.5	3.30 ± 0.083		0.396 ± 0.010
AS400/Pho8vac		8.11 ± 0.18	125.3 ± 4.4	3.75 ± 0.075		0.450 ± 0.010

^*^Results of three independent tests.

^**^Results of two independent tests.

### Overexpression of the vacuolar form of alkaline phosphatase in AS400 industrial strain

**Figure 3:**
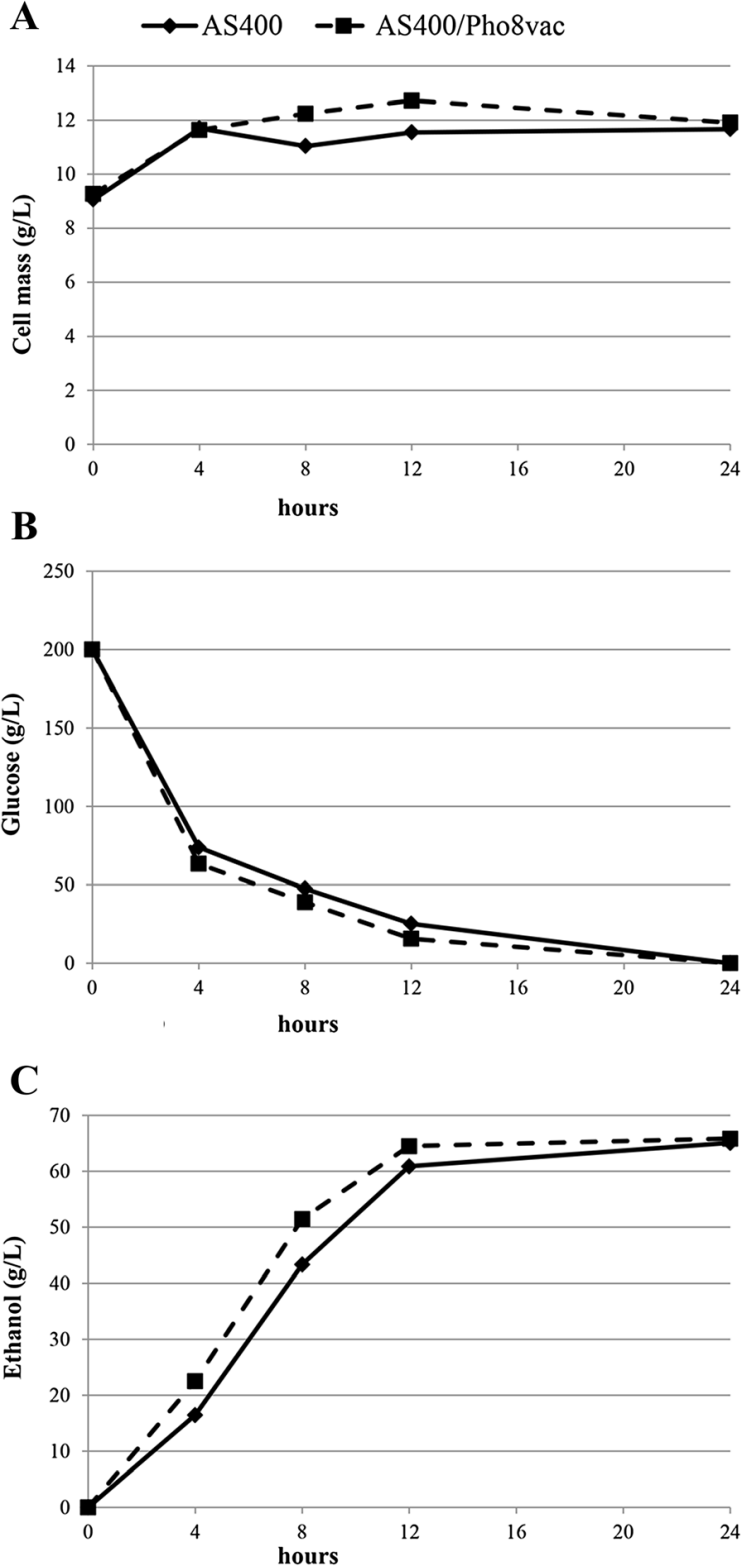
**Growth, glucose consumption and ethanol production during alcoholic fermentation of recombinant strains overexpressing vacuolar form of alkaline phosphatase.** Strains were cultured under semianaerobic conditions in YNB medium with 200 g/L D-glucose. Cultures were incubated at 30°C, shaking at 120 rpm using. AS400 – WT, recipient strain. AS400/Pho8vac – strain containing vector pUC57-delta1_2-ADHpr-PHO8-CYCt-kanMX. **A** – biomass accumulation (g of biomass/L of medium); **B** – glucose consumption (g of glucose/L of medium); **C** – ethanol production (g of ethanol/L of medium).

The vacuolar form of alkaline phosphatase was also expressed in the industrial ethanol-producing strain AS400. The plasmid pUC57-delta1_2-ADHpr-PHO8-CYCt-kanMX was used for transformation of the strain AS400. Selected transformants AS400/Pho8vac had 2.8-fold increase in the specific activity of alkaline phosphatase as compared to the parental strain AS400 (Table 2). The recombinant strain AS400/Pho8vac with the highest specific activity of alkaline phosphatase and the parental AS400 strain were tested for the efficiency of alcoholic fermentation in a mineral YNB medium supplemented with 20% glucose. Biomass and ethanol accumulation was measured in transformant AS400/Pho8vac and the parental strain AS400 (Figure 3). Based on the results, it was determined that the transformant AS400/Pho8vac accumulated 6% more ethanol relative to parental strain during fermentation producing 5.37 g/L/h of ethanol versus 5.07 g/L/h for AS400 strain (Figure 3). Due to slight difference in the biomass accumulation and complete glucose consumption during fermentation, differences in ethanol production between parental and recombinant strains may be caused by the decrease of byproducts accumulation like glycerol, acetate or reduced amount of reserve carbohydrates. The recombinant strain AS400/Pho8vac was also tested for the efficiency of alcoholic fermentation in a CSL medium supplemented with hydrolysed corn meal. This strain was shown to have substantially increased productivity of ethanol synthesis (3.75 g/L/h) as compared to that of the parental strain (3.3 g/L/h) (Table 2). Ethanol yield per gram of consumed glucose was found to be increased 13.6% (Table 2). Measurement of the productivity of ethanol synthesis per biomass was not carried out in CSL medium as it contains many small insoluble particles which hamper cell biomass measurement using direct dry weight or by optical density analyses. The recombinant strains tested were all also characterized by substantially lower intracellular ATP level relative to the parental strain AS400 (Table 2). We do not exclude the possibility that the drop in intracellular ATP level may have a negative impact on biomass accumulation during aerobic large-scale cultivation, but we assume that this effect will be insignificant, since biomass accumulation of recombinant strain AS400/Pho8vac didn’t differ substantially from the parental strain AS400 in the experiments we carried out (Figure 3A).

#### ***Overexpression of the cytosolic form of alkaline phosphatase in BY4742***

The results demonstrate that the overexpression of vacuolar alkaline phosphatase leads to an increase in ethanol production during alcoholic fermentation of glucose in laboratory and industrial strains of *S. cerevisiae*. This is due to the accelerated hydrolysis of substrate via increased activity from overexpression of vacuolar alkaline phosphatase. The target substrates are polyphosphates which are known to be the natural substrates of alkaline phosphatase [21]. As the synthesis of polyphosphates requires ATP, an increase in alkaline phosphatase activity may lead to a futile cycle of ATP consumption which enhances need for ATP to replenish again the vacuolar polyphosphate pool [22]. We decided to check the effects of overexpression of the truncated forms of alkaline phosphatase which lack putative vacuolar targeting signal. A recombinant strain expressing truncated version of *PHO8* gene was constructed. Alkaline phosphatase is synthesized as an inactive precursor containing a C-terminal propeptide that is afterwards cleaved from the protein in yeast vacuoles in a Pep4-dependent manner [19]. During vacuolar delivery, which shares the early stages with the secretory pathway, the precursor form of this enzyme is glycosylated in the endoplasmic reticulum. It has also been shown that the active form of alkaline phosphatase binds with the metal co-factor zinc in the vacuole rather than in earlier compartments of the secretory pathway [23]. A truncated form of this enzyme was constructed which lacks 60 initial amino acids that are responsible for transmembrane protein delivery; and additionally 22 terminal amino acids, that represent the C-terminal propeptide that is normally cleaved from the protein in the vacuole [19,23]. The DNA fragment containing the expression cassette of the truncated *PHO8* gene in part with selective marker flanked by δ sequences were used for transformation of BY4742 strain of *S. cerevisiae*. The specific alkaline phosphatase activity of the selected recombinant strain BY4742/Pho8cyt was assayed and compared with that of the strain BY4742/Pho8vac. The intracellular alkaline phosphatase activity in the strain BY4742/Pho8cyt was at the same level as in the strain BY4742/Pho8vac overexpressing the intact form of *PHO8* (Table 2).

Growth kinetics of the constructed recombinant strains was measured. An equal amount of cellular biomass (0.015 g/L) of wild-type strain and recombinant strains were inoculated into the mineral media containing 10% glucose as a carbon source. The strain with truncated *PHO8* form was characterized by a longer lag-phase during the first day of cultivation (not shown), with lowered biomass accumulation and a reduced growth rate (Table 2). This recombinant strain (BY4742/Pho8cyt) had the highest specific activity of cytosolic form of alkaline phosphatase showed a decrease in the intracellular ATP level (Table 2).

Recombinant strain BY4742/Pho8cyt has produced significantly less ethanol during glucose alcoholic fermentation relative to recombinant strains with overexpressed native alkaline phosphatase and the wild-type strain BY4742, i.e. only about 0.42 g/L/h or 0.18 g/g of biomass/h (Table 2). We hypothesize that the cytosolic form of alkaline phosphatase decreases not only the level of ATP but also that of other sugar phosphate intermediates as well as regulators like fructose-2,6-bisphosphate, leading to detrimental effect on ethanol production. However, confirmation of this hypothesis requires additional experiments which are in progress.

## Conclusion

This study established a new successful approach to increasing ethanol production from glucose in *S. cerevisiae* by homologous overexpression of *PHO8* gene due to multiple genomic insertion events. The overexpression of intact *PHO8* gene encoding vacuolar form of alkaline phosphatase led to decline in intracellular ATP level and biomass accumulation and up to 13% increase in ethanol productivity. By comparison, the overexpression of truncated form of *PHO8* gene encoding presumably cytosolic form of alkaline phosphatase caused a significant drop both in biomass accumulation and ethanol production. In conclusion, the overexpression of the intact vacuolar form of alkaline phosphatase results in overproduction of ethanol in laboratory and industrial strains of *S. cerevisiae*.

## Methods

### Strains, media, growth and fermentation conditions

The *S. cerevisiae* strain BY4742 (*MAT*α, *his3*Δ*1*, *leu2*Δ*0*, *lys2*Δ*0*, *ura3*Δ*0*; [24] and an industrial strain AS400 (obtained from Archer Daniels Midland Company, Decatur, IL, USA) (Table 1) were used for the expression of the intact or truncated version of *PHO8* ORF coding for alkaline phosphatase. For selection of yeast transformants on YPD, 200 mg/L of geneticin was added. When required, histidine (20 mg/L), leucine (60 mg/L), lysine (20 mg/L), or uracil (20 mg/L) were added.

*S. cerevisiae* strains were incubated at 30°C. Yeast strains were maintained in rich YPD (1% yeast extract, 1% peptone and 2% glucose) or mineral YNB (0.67%, yeast nitrogen base without amino acids, DIFCO, 0.5% ammonium sulfate, 2% glucose) media. For ethanol fermentation, all strains were tested with a YNB medium that was supplemented with 10% glucose or by using a medium containing corn steep liqour (CSL) supplemented with hydrolyzed corn meal as the primary carborn source.

The corn steep liquor (CSL) medium was prepared by combining two solutions that were prepared as follows. Solution 1 was prepared by adding 200 g of corn meal (Melvit, Poland) was mixed with 800 ml of water and pH adjusted to 6.0 using NaOH. The enzyme alpha-amylase (Liquizyme SC) was added at a rate of 0.1 units of per gram of meal and the slurry was liquefied by heating the mixture to 80˚C for 30 minutes. To prepare Solution 2, 63 ml of CSL Concentrate (containing ~50% dry solids) was mixed with 137 ml of deionized water. The two solutions were autoclaved, cooled, combined and mixed. The enzyme glucoamylase (Liquizyme SC) was added at a rate of 0.2 units of glucoamylase per gram of meal to sterile flasks and the flasks were incubated at 28°C. Glucose concentration was measured in control flasks without yeast inoculation at 180–200 g/L in the CSL medium prepared.

Cells of BY4742 strain and its *PHO8-*expressing derivatives were grown in 100 ml of YPD medium in Erlenmeyer flasks (bottle size – 300 ml) overnight and then used to inoculate a 20 ml of YNB medium with 10% glucose in 50 ml Erlenmeyer flasks. An initial biomass concentration of 1.2 g (dry weight)/L was used for fermentation. Fermentation was carried out at a temperature of 30°C with limited aeration using a gyratory shaker at a setting of 120 revolutions/min.

For the *S. cerevisiae* AS400 strain and its *PHO8-*expressing derivatives, both YNB medium with 20% glucose and CSL medium supplemented with hydrolyzed meal were used in fermentation tests. An initial biomass concentration of 10 g (dry weight)/L was used for fermentation as this is the cell density normally used in industrial ethanol fermentation [25]. Strains were incubated under semi-anaerobic condition at 34˚C for 2 days and samples were taken every 24 hours.

The *E. coli* DH5α strain (Φ80d*lacZ*ΔM15, *recA*1, *endA*1, *gyrA*96, *thi*-1, *hsdR*17(r_K_^-^, m_K_^+^), *supE*44, *relA*1, *deoR*, Δ(*lacZYA-argF*)U169) was used as a host for propagation of plasmids. Strain DH5α was grown at 37°C in LB medium as described previously [26]. Transformed *E. coli* cells were maintained on a medium containing 100 mg/L of ampicillin. The chromogenic substrates X-gal and IPTG (Fermentas, Vilnius, Lithuania) were used according to the manufacturer specifications.

#### DNA manipulations

Genomic DNA from *S. cerevisiae* strains was isolated using the Wizard^®^ Genomic DNA Purification Kit (Promega, Madison, WI, USA). Plasmid DNA from *E. coli* was isolated using the Wizard^®^*Plus* SV Minipreps DNA Purification System (Promega). Taq and High Fidelity polymerase mix, T4 DNA ligase, T4 DNA polymerase and restriction enzymes were used according to recommendation of supplier (Fermentas). *S. cerevisiae* transformation was performed by standard protocol [26].

#### Plasmid construction

**Figure 4:**
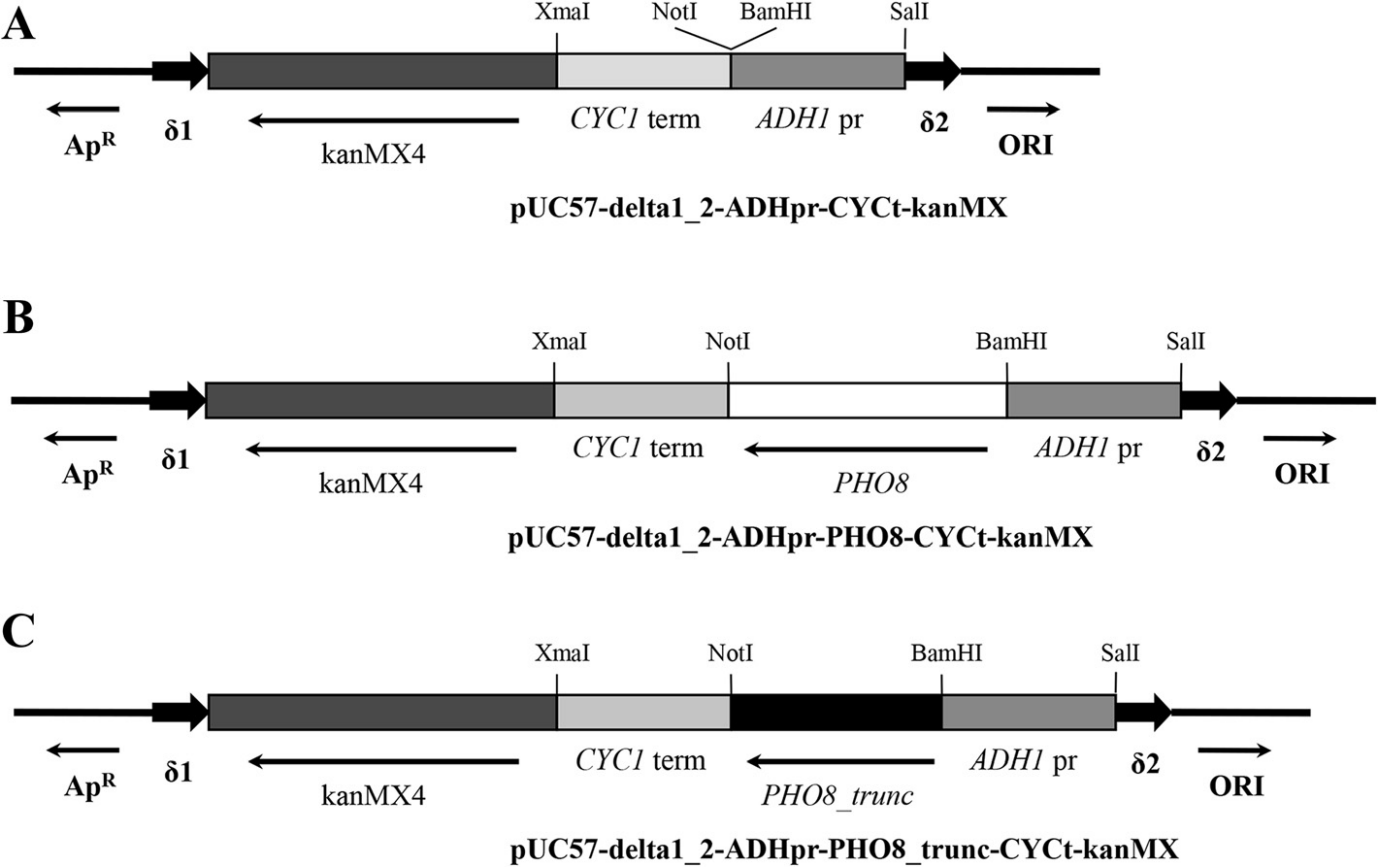
**Linear schemes of plasmids used in this study: pUC57-delta1_2-ADHpr-CYCt-kanMX (A), pUC57-delta1_2-ADHpr-PHO8-CYCt-kanMX (B), pUC57-delta1_2-ADHpr-PHO8_trunc-CYCt-kanMX (C).** δ elements are shown as thick black arrows. *ADH1* promoter, *CYC1* terminator and *kanMX4* gene are shown as chessboard-alike, doted and diagonal-hatched boxes, respectively. Unmodified *PHO8* gene ORF is shown as white box and truncated *PHO8* gene ORF is shown as black doted box. Origin of replication ORI and ampicillin resistance gene (*bla*) are shown as thin arrows.

*Expression cassette preparation.* 154 bp 5’- and 180 bp 3’-parts of *S. cerevisiae* YJRWdelta12 sequence were amplified from genomic DNA of *S. cerevisiae* strain BY4742 using pairs of primers SM16/SM17 and SM18/SM19 (Table 3). Both delta sequences were fused via overlap PCR using primers SM16 and SM19, digested with EcoRI and HindIII and cloned into EcoRI/HindIII-linearized plasmid pUC57. The constructed plasmid was designated pUC57-delta1_2. Fragments of DNA having 807 bp and 269 bp bearing promoter of *ADH1* gene encoding alcohol dehydrogenase and terminator of *CYC1* gene encoding cytochrome С, were amplified from genomic DNA of BY4742 by pairs of primers Ко419/Ко420 and Ко453/Ко454, respectively. The *ADH1* promoter and *CYC1* terminator were fused via overlap PCR using primers Ко419 and Ко454 and SalI/XmaI cloned into the corresponding sites of the plasmid pUC57-delta1_2 resulting in the plasmid pUC57-delta1_2-ADHpr-CYCt. A 1470 bp DNA fragment corresponding to the selective marker *kanMX* providing resistance to geneticin was cut out from the plasmid pRS303K [27] with restriction endonucleases SacI and SmaI, blunt-ended and cloned into XbaI-digested and blunted plasmid pUC57-delta1_2-ADHpr-CYCt yielding the plasmid pUC57-delta1_2-ADHpr-CYCt-kanMX (Figure 4A).

**Table 3:** DNA oligonucleotides used in this study

**Primer names**	**Sequence (5’-3’) restriction sites are italic**	**Restriction sites**
SM16	CCG*GAATTC*^a^*GACGGGCAGTC*^b^TGTTGGAATAGAAATCAACTATC	EcoRI^a^, AhdI^b^
SM17	CATCATTTTATATGTTTATATTCA*TCTAGA*^a^*CCCGGG*^b^*GTCGAC*^c^ TTGATCCTATTACATTATCAATCC	XbaI^a^, XmaI^b^, SalI^c^
SM18	GGATTGATAATGTAATAGGATCAA*GTCGAC*^a^*CCCGGG*^b^*TCTAGA*^c^TGAATATAAACATATAAAATGATG	SalI^a^, XmaI^b^, XbaI^c^
SM19	CCC*AAGCTT*^a^*GACGGGCAGTC*^b^TGAGAAATATGTGAATGTTGAG	HindIII^a^, AhdI^b^
SM28	CGC*GGATCC*^a^ATGTCTGCATCACACAAGAAGAAGAATGTC	BamHI^a^
SM29	TTT*GCGGCCGC*^a^TCAATCTGATGTGTGTTTGGTGTCCCTAATC	NotI^a^
Ko419	CGC*GTCGAC*^a^TTAATTAAAGTCCAATGCTAG	SalI^a^
Ko420	GATATCGACAAAGGAAAAGGG*GCGGCCGC*^a^*GGATCC*^b^*CTCGAG*^c^TGTATATGAGATAGTTGATTG	NotI^a^, BamHI^b^, XhoI^c^
Ko453	CAATCAACTATCTCATATACA*CTCGAG*^a^*GGATCC*^b^*GCGGCC GC*^c^CCCTTTTCCTTTGTCGATATC	XhoI^a^, BamHI^b^, NotI^c^
Ko454	CCC*CCCGGG*^a^GCAAATTAAAGCCTTCGAGC	XmaI^a^
Ko508	CGC*GGATCC*^a^ATGATGACTCACACATTACCAAGC	BamHI^a^
Ko509	TTT*GCGGCCGC*^a^TCAGTTGGTCAACTCATGGTAGTATTC	NotI^a^

^a,^^b,^^c^Restriction sites with corresponding restriction enzyme.

*Plasmid for expression of native PHO8*. A 1701 bp DNA fragment bearing the ORF of *PHO8* gene coding for unspecific alkaline phosphatase was amplified from genomic DNA of the BY4742 strain using primers Ko508 and Ko509. This fragment was digested with BamHI and NotI and subcloned into BamHI/NotI digested plasmid pUC57-delta1_2-ADHpr-CYCt-kanMX. This plasmid was designated pUC57-delta1_2-ADHpr-PHO8-CYCt-kanMX (Figure 4B).

*Plasmid for expression of truncated version of PHO8 gene*. The cytosolic form of Pho8 lacking 60 initial amino acids which are responsible for transmembrane protein delivery; and 22 terminal amino acids composing the C-terminal propeptide that is normally cleaved from the protein in vacuole, was isolated. A 1452 bp DNA fragment corresponding to this truncated form, was amplified from genomic DNA of BY4742 strain using primers SM28 and SM29, digested with endonucleases BamHI and NotI and cloned into BamHI/NotI digested plasmid pUC57-delta1_2-ADHpr-PHO8-CYCt-kanMX. The constructed plasmid was designated as pUC57-delta1_2-ADHpr-PHO8_trunc-CYCt-kanMX (Figure 4C).

### Selection of *S. cerevisiae* transformants

The vectors containing native or modified versions of the *PHO8* gene were digested with the AhdI. AhdI-fragments containing expression cassettes and selective marker flanked by δ-sequences, were eluted from an agarose gel and used for transformation of *S. cerevisiae* strains BY4742 and AS400. The transformants were selected on a solid YPD medium supplemented with 200 mg/L of geneticin. The selected transformants were stabilized by alternating cultivation in non-selective and selective media and examined by diagnostic PCR using a forward primer Ko419 specific to the *ADH1* promoter and a reverse one Ko509 specific to *PHO8* gene.

### Biochemical methods and analyses

The specific activity of alkaline phosphatase was assayed in cell free extracts using the chromogenic substrate p-nitrophenyl phosphate as described elsewhere [18]. The assay was repeated three times and the measurement reported is an average of these determinations. The numbers reported use one unit of alkaline phosphatase activity as the amount of enzyme that liberates 1 μmol of p-nitrophenol per min under the assay conditions.

Measurements of ATP were carried out following extraction of this metabolite from the *S. cerevisiae* cells was using boiling ethanol and subsequent evaporation [28]. In order to optimize ATP assay condition for ethanol extraction, a wide range of yeast biomass from 2 to 15 mg was tested. Reproducible results were obtained during ATP extraction with 8–10 mg of *S. cerevisiae* cells using 1 ml of boiling ethanol. Extracted ATP was measured by coupled enzymatic reactions with hexokinase and glucose-6-phosphate dehydrogenase [29].

The concentration of ethanol in fermentation media was determined using alcohol oxidase/peroxidase-based enzymatic kit “Alcotest” [30,31]. The biomass was determined using turbidity with a Helios Gamma spectrophotometer (OD, 600 nm; cuvette, 10 mm) with gravimetric calibration. Glucose concentration was determined using the “Diagluc” assay kit (UBT, Lviv) [30]*.* All samples were assayed at least twice to ensure results are reproducible.

## Competing interests

The authors declare that they have no competing interests.

## Authors’ contributions

MVS carried out gene engineering manipulations, enzyme activity assays and alcoholic fermentations. KVD participated in design of cloning and strain construction, analyzed the date and co-drafted the manuscript. CAA participated in plasmids sequencing, commented and approved the manuscript. AAS provided guidance and suggestions for experimental design and edited the manuscript. All authors have read and approved the manuscript.
